# Approaches to Improve Macromolecule and Nanoparticle Accumulation in the Tumor Microenvironment by the Enhanced Permeability and Retention Effect

**DOI:** 10.3390/polym14132601

**Published:** 2022-06-27

**Authors:** Victor Ejigah, Oluwanifemi Owoseni, Perpetue Bataille-Backer, Omotola D. Ogundipe, Funmilola A. Fisusi, Simeon K. Adesina

**Affiliations:** 1 Department of Pharmaceutical Sciences, College of Pharmacy, Howard University, Washington, DC 20059, USA; victor.eligah@bison.howard.edu (V.E.); oluwanifemi.owoseni@bison.howard.edu (O.O.); perpetue.batailleba@bison.howard.edu (P.B.-B.); omotola.ogundipe@bison.howard.edu (O.D.O.); funmilolaadesodun.fi@howard.edu (F.A.F.); 2 Faculty of Pharmacy, Obafemi Awolowo University, Ile-Ife 220005, Nigeria

**Keywords:** enhanced permeability and retention effect, nanotechnology, tumor microenvironment, Zwitterionic polymers, synthetic microbe, macrophages, liposomes

## Abstract

Passive targeting is the foremost mechanism by which nanocarriers and drug-bearing macromolecules deliver their payload selectively to solid tumors. An important driver of passive targeting is the enhanced permeability and retention (EPR) effect, which is the cornerstone of most carrier-based tumor-targeted drug delivery efforts. Despite the huge number of publications showcasing successes in preclinical animal models, translation to the clinic has been poor, with only a few nano-based drugs currently being used for the treatment of cancers. Several barriers and factors have been adduced for the low delivery efficiency to solid tumors and poor clinical translation, including the characteristics of the nanocarriers and macromolecules, vascular and physiological barriers, the heterogeneity of tumor blood supply which affects the homogenous distribution of nanocarriers within tumors, and the transport and penetration depth of macromolecules and nanoparticles in the tumor matrix. To address the challenges associated with poor tumor targeting and therapeutic efficacy in humans, the identified barriers that affect the efficiency of the enhanced permeability and retention (EPR) effect for macromolecular therapeutics and nanoparticle delivery systems need to be overcome. In this review, approaches to facilitate improved EPR delivery outcomes and the clinical translation of novel macromolecular therapeutics and nanoparticle drug delivery systems are discussed.

## 1. Introduction

Improved clinical outcomes in the treatment of some cancers have not been observed despite tremendous efforts in the development of new chemotherapeutic agents. This is largely due to the failure to deliver these agents selectively to tumors and the lack of precision in targeting cancer cells [1]. This poor targeting to tumors greatly increases the propensity for debilitating off-target consequences and minimizes the magnitude of therapeutic efficacy. Innovations in the field of nanotechnology, immunology, chemistry, and pharmaceutical technology have led to the development of a variety of drug delivery systems aimed at improving the plasma half-life, biodistribution, and the target-site accumulation of chemotherapeutic drugs [2,3].

Nanoparticles for drug delivery are carrier systems in the nanometer size range, composed of biocompatible and biodegradable natural and/or synthetic polymers, self-assembled lipids, or inorganic materials, capable of carrying payloads such as small molecule therapeutics, peptides, proteins, or nucleic acids and delivering their cargoes in a controlled manner at the target site [4]. These delivery systems have shown promise in preclinical trials, and some nanoparticles, such as Doxil^TM^ (doxorubicin/liposome) and Abraxane^TM^ (paclitaxel/albumin), designed to modify the pharmacokinetics of existing chemotherapeutic agents, are already routinely used in clinical practice [2,5].

The enthusiasm around nanocarrier systems has led to a plethora of publications demonstrating the efficacy of different constructs in a variety of animal cancer models; however, a meta-analysis of published studies [6] revealed that in the past decade, only about 0.7% tumor site accumulation was achieved for particles with sizes below 100 nm in solid tumors. While this may appear to be low, nanomedicines have demonstrated substantially higher delivery efficiencies than most conventional chemotherapeutic formulations in relative terms [7,8]. It has been reported that the dense extracellular matrix, high interstitial fluid pressure, and non-uniform blood perfusion limit nanoparticle accumulation in solid tumors [7,9]. The preferential accumulation of drug-loaded nanoparticles in neoplastic tissues is referred to as passive targeting [9,10]. Passive targeting is facilitated by the enhanced permeability and retention (EPR) effect [11], i.e., the mechanism by which high molecular weight drug carriers accumulate in the tumor microenvironment (TME) due to increased vascular permeability and the nanometer size of nanoparticles [12]. The TME also possesses impaired lymphatic drainage that prevents the efficient removal of these macromolecules or nanoparticles, thus enhancing their retention within neoplastic tissues [13,14,15].

The concept of EPR originated from a landmark study in 1986 by Matsumura and Maeda on the mechanisms of the tumoritropic accumulation of proteins and chemotherapeutic agents [16]. In this study, an increase in the accumulation and uptake of a derivatized styrene-maleic acid polymer loaded with neocarzinostatin (SMANCS) was observed in tumor cells relative to the native neocarzinostatin. SMANCS has a molecular size of 16 kDa and can bind serum albumin (67 kDa) to become a larger molecule [17]. The superior accumulation of SMANCS in tumor tissues offered prolonged duration of action and increased therapeutic efficacy. The authors opined that EPR is made possible by an increased vascular permeability, a dysfunctional lymphatic drainage system, and the relative size of nanoparticles. They showed that very small molecules will traverse biological barriers easily, while larger molecules like nanoparticles may be filtered through highly vascularized and permeable barriers. The dependence of EPR on molecular size was further demonstrated via the encapsulation of SMANCS in liposomes (ethiodized poppyseed oil); an outcome that stimulated research on the use of liposomes as chemotherapeutic drug delivery systems [12].

In contrast to tumor blood supply, blood supply to healthy tissues is well organized with tight endothelial junctions. Healthy tissues also possess a functional lymphatic drainage system that rids the extracellular matrix of nonresident molecules. The difference between healthy and neoplastic blood supply forms the basis of the selectivity inherent in the EPR effect. The effectiveness of EPR has been validated [18,19] and has led to the discovery and development of carrier-based therapeutic products [20] that are currently in clinical use. With advances in the field of nanoparticle drug delivery, it has become apparent that the therapeutic efficacy of passively targeted nanomedicines is vastly influenced by the heterogeneity of the intensity of the EPR effect within a tumor, at different stages of a tumor, and among individual tumors [21], as well as other physiological barriers including the reticuloendothelial system.

In this review, we will discuss the principle of EPR as a function of the features of the tumor microenvironment. We will also summarize the challenges of EPR-based passive tumor targeting. Our goal is to explore strategies and approaches for enhancing the accumulation of macromolecules and nanoparticles within the tumor microenvironment for improved therapeutic outcomes.

## 2. Principle of EPR

**Figure 1:**
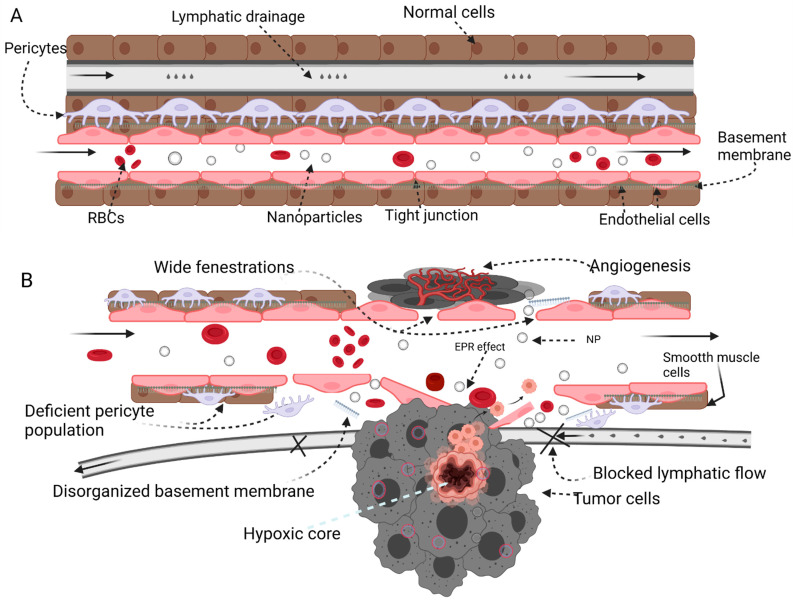
Comparison of the microenvironment of (**A**) healthy and (**B**) tumor tissues. The tumor microenvironment shows the disorganized components (hypoxic core, blocked lymphatic drainage, deficient pericyte population, disorganized basement membrane and wide fenestration) that are exploited for enhancing EPR effect. Created with BioRender.com Accessed on 25 July 2021.

The unique vascular physiology of solid tumors is the basis for the EPR effect. First, the microvasculature in solid tumor (Figure 1) tissues lacks basement membrane support, making it unresponsive to physiological cues and stimuli that regulate blood flow [10]. Second, rapid metabolism in tumor cells leads to a high demand for nutrients and oxygen, which drives angiogenesis at high rates and prevents adequate vessel maturation [22]. Taken together, these factors create a hyperpermeable state with fenestrations in the endothelial cell lining of newly formed tumor blood vessels [23]. The leaky, heterogenous, and disorganized nature of neoplastic blood vessels allows the escape of macromolecules and nanoparticles into tumor tissues. In addition, nanoparticle entrapment and retention in the tumor interstitium occur because of the tumor’s dysfunctional lymphatic drainage system, preventing proper drainage [3].

Thus, tumor-selective anticancer drug delivery can be achieved by attaching, conjugating, or encapsulating low-MW antineoplastic drugs to macromolecular carriers (dendrimers, liposomes, polymers, and micelles) in single or multiple-drug formulations [24].

The EPR effect-mediated tumor accumulation of macromolecules and nanoparticles has been demonstrated in several studies [25] with carrier molecular sizes of more than 40 kDa and particle sizes of 6–8 nm or larger [26]. For passive targeting via the EPR effect, several criteria must be met. First, the macromolecular carrier must remain in systemic circulation for a considerable length of time because the accumulation at the tumor site via the EPR effect is a time-dependent occurrence [18]. Second, macromolecular carriers must possess critical quality attributes such as: (i) appropriate size to allow extravasation and accumulation in tumors via the wide fenestrations found only in tumor blood supply; (ii) an ideal surface charge to avoid opsonin aggregation; (iii) an optimum surface chemistry that allows tissue penetration; (iv) stability of macromolecule integrity to avoid nonspecific release; and (v) biocompatibility [24]. Finally, the nanoparticles must have a uniform size distribution to avoid aggregation due to a high surface to volume ratio which predisposes to recognition by the reticuloendothelial system, especially the Kupffer cells, liver sinusoidal endothelial cells, and liver stellate cells that are responsible for the elimination of 30–99% of injected nanoparticles and macromolecules [27].

## 3. Essential Considerations for EPR Effect

The translation of various macromolecular and nanocarrier constructs from successful preclinical experiments to clinical practice has been largely unsuccessful, despite all the efforts made to optimize nanoparticle design to promote active or passive targeting [28]. A meta-analysis by Wilhelm [6] reported that for all the published articles evaluated, only 0.7% of the injectable dose (ID) on average was found in the tumor. Of all the parameters analyzed, only hydrodynamic size < 10 nm (0.7%; *p* = 0.0001), neutral zeta potential (0.7%; *p* = 0.0068), spherical shape (0.8%; *p* = 0.00479), and orthotopic tumor models (1.1%; *p* = 0.001) correlated significantly to the highest percentage delivered. Upon stratification to determine delivery efficiency based on type of nanomaterials, organic nanomaterials showed significantly superior efficiency compared to inorganic materials for the parameters mentioned above. Therefore, it is logical to optimize these attributes in organic nanocarriers to engender greater delivery efficiency and increase accumulation of macromolecules and nanoparticles within the TME.

The differences that exist between tumor xenografts in mice models and tumors in man must be considered in translating preclinical successes to clinical settings [2,29,30,31]. Most cell line grafts in animal models are much less heterogeneous than human tumors because the experimental models are usually standardized—same genetic background, origin, and age—to allow valid statistical analyses. Human tumors, on the other hand, can be highly heterogeneous, i.e., ranging from 1 mm to as large as 100 mm or more, and are observed in individuals of different ages, lifestyles, and genetic backgrounds. In addition, the possibility that currently used xenograft models are not suitable for direct comparison and extrapolation to human tumors must also be considered [2,20,32,33]. For instance, the rate of development of tumors in animal models is faster than in humans; this rapid rate of tumor growth results in accelerated angiogenesis, leading to unusually disorganized vascular walls which are amenable to EPR [3]. Similarly, the rate of metabolism in mice is faster than that in humans; this allows a more aggressive dosing of macromolecules [34].

The total amount of nanoparticles required to deliver the desired payload is another factor to be taken into consideration. Data from the study by Wilhelm et al. shows that to achieve IC_50_ in a tumor volume of 0.5 cm^3^ for a mouse of about 20 g body weight, a total of 1.2 × 10^12^ nanoparticles, or a dose of 6.5 mg kg^−1^, must be injected, provided the nanoparticles encapsulate 20 wt% of the drug. They also posited that an increase in this dose to a total of 2.8 × 10^12^ nanoparticles or 15.7 mg kg^−1^ will be necessary if drugs are loaded on the particle surface at a surface density of one drug molecule per nm^2^. This dose is feasible for preclinical administration in mice. Translating this to man on similar metrics would require a dose of 2.7 × 10^14^ drug-encapsulated nanoparticles, or 6.4 × 10^14^ surface-loaded nanoparticles, based on the surface-area dosing strategy [35]. This is a challenging proposition, as it would require scaling up production of nanoparticles, which may lead to issues of colloidal instability, aggregation, short shelf life, systemic toxicity, and poor bioavailability due to elimination by the reticuloendothelial system [6]. Other limitations in the translation of experimental results of EPR in animal models to the clinic include differences in genetics, immunology, syngeneic attributes, and non-orthotopic tumor grafting [36].

Additional differences between tumor xenografts in mice models and tumors in man are seen in the relative size of tumors to the host body weight. Mice tumors are usually grown to more than 10% of the animal’s total body weight before treatment is administered. The high tumor volume relative to the total body weight in mice allows for significant contact with circulating drug loaded nanoparticles, leading to better efficacy outcomes [3]. In contrast, some human tumors constitute just about 0.005% of the total body weight of a 70 kg man. Therefore, for a chance to significantly encounter circulating drug loaded nanoparticles, a tumor requires considerable exposure, i.e., of an average of ten days or more [3]. Hence, a loaded nanoparticle has a higher propensity to There appear to be a plethora of challenges in achieving adequate accumulation of nanoparticles in the TME based on the essential factors discussed above. Therefore, to successfully translate preclinical efforts to human subjects, the fundamental strategy will be to improve the delivery efficiency of nanoparticles to tumors [6]. Part of this strategy would involve creating tumor models that would properly replicate the human TME to produce outcomes that are reproducible post-translation. To achieve this, some studies have attempted to slow down the rate of angiogenesis in mice by using anti-angiogenic factors to encounter and extravasate into a mice xenograft tumor by EPR than it does a human tumor produce vessels that are less permeable to large nanoparticles [22]. Other novel experimental tumor models, including the use of laboratory methods, organ-on-a-chip methods, ex vivo systems, and dynamic organoids are becoming increasingly popular [37].

The meta-analysis by Wilhelm et al. has not gone unchallenged. A recent publication [23] asserted that the analysis did not take into consideration important factors such as tumor size, effectiveness of drug delivery at target site, and tumor heterogeneity. They observed that the analysis focused on percentage injected dose while ignoring the ratio of drug concentration in the tumor to that in blood. Similarly, another study [38] performed a reanalysis on the same data set used by Wilhelm et al. and concluded that based on traditional pharmacokinetic (PK) evaluation, the %ID in tumor was poorly correlated with standard PK metrics that describe nanoparticle tumor delivery (AUC_tumor_/AUC_blood_ ratio) and is only moderately associated with maximal tumor concentration. The author proposed that a better interpretation of the finding of Wilhelm et al. should be that an average of 0.67% of ID was found in the tumor per hour interval throughout the entire PK evaluation period. Using the same dataset and based on the more appropriate AUC_tumor_/AUC_blood_ ratio metric, Price et al. showed that the exposure of tumors to overall plasma nanoparticles (AUC_blood_) was 76.12%, i.e., a 100-fold greater value than that based on %ID. If this were the case, then it is expected that preclinical trials should translate successfully to clinical settings, but that is not the experience so far. Therefore, the imperative to explore further and improve EPR cannot be overemphasized.

## 4. Approaches and Techniques to Improve EPR Effect

### 4.1. Modification of Physicochemical Properties of Nanoparticles and Macromolecules

#### 4.1.1. Particle Size

Particle size is a fundamental characteristic of nanoparticles that significantly influences the efficiency of tumor targeted delivery systems with respect to circulation, biodistribution, tumor penetration/accumulation, and cellular uptake [39]. The EPR effect is substantially dependent on the particle size of nanoparticles. In general, small molecules or particles with sizes less than the renal glomerular filtering threshold of 40 kDa or 6–8 nm are largely removed via renal excretion or via the liver by the stellate cells, and are eliminated in urine or feces [40]. The optimum nanoparticle size is largely dependent on the type of tumor; a small nanoparticle size does not necessarily translate to improved tumor delivery via the EPR effect [39]. It has been demonstrated that small size is critical to tumor tissue penetration [20]; however, small-sized macromolecules and nanoparticles are easily extruded by the interstitial pressure within the tumor environment [41]. On the flip side, large molecules are poor at penetrating tumor cells, but when effectively delivered, are more likely to be retained [42].

The large size of nano-based drugs, therefore, plays a critical role in EPR-dependent drug accumulation in tumors, because they are less likely to be eliminated [24]. Most nanomedicines are designed in the range of 10–200 nm in diameter, but even particle sizes up to 1–2 μm (bacteria cells) could also accumulate in tumor cells via the EPR while sparing healthy tissues [43]. Such macromolecules show prolonged circulation time (increased t_1/2_) and high area under the concentration-time curve (AUC) in plasma, thus enabling gradual permeation and accumulation within tumor tissues [19].

Striking the right balance between tissue permeation and retention of nanoparticles has been a subject of research in the recent past. In one study, an evaluation of the permeation and retention ability of nanoparticles of different sizes showed that 60% of nanoparticles in the range of 100–200 nm circulated longer in plasma compared to 20% of nanoparticles with sizes less than 50 nm and 20% of nanoparticles greater than 250 nm. Another study reported a four-fold uptake of nanoparticles between 100–200 nm compared to nanoparticles with sizes less than 50 nm and greater than 300 nm [44]. These results clearly show that nanoparticles with small particle sizes may not be ideal in providing adequate tumor accumulation in some circumstance, considering how quickly they could be extruded from the tumor microenvironment.

A strategy designed to strike the balance between tissue penetration and retention of nanoparticles has been demonstrated in the formulation of large gelatin-based nanoparticles of 100 nm that can be degraded by tumor-associated matrix metalloproteinases (MMPs) to 10 nm upon extravasation to promote tissue penetration [45].

#### 4.1.2. Surface Charge

The surface charge of nanoparticles and macromolecules is a critical physicochemical property that should be tailored to prolong circulation half-life and enhance accumulation in tumors via the EPR effect [24]. This is because surface charge is associated with solubility, aggregation, biocompatibility, and the ability of nanoparticles to move across biological barriers [33]. The vascular endothelial luminal surface and membranes of the cells of the liver and spleen have very high negative charge, so nanoparticles with positive charges or cationic polymers are easily bound by these surfaces, resulting in a rapid decrease in plasma concentration due to removal by the reticuloendothelial system (RES) [46]. Besides, strong electrostatic interaction between cell membranes and positively charged nanoparticles has been shown to cause cytotoxicity [47]. That notwithstanding, some studies have shown that positively charged nanoparticles can still achieve good cell uptake depending on the type of cell. For instance, positively charged nanoparticles were used to induce a significant adaptive immunologic response to a pulmonary vaccine compared to a negatively charged control. Positively charged nanoparticles were more associated to dendritic cells that are required for adaptive immunity, unlike negatively charged nanoparticles, that were more associated with alveolar macrophages [48]. In another instance, cationic nanoparticles have been shown to attract proteins which form complexes that enhance cell uptake. The mechanism is largely due to the nature of the receptors found on specific cells. The proteins that adsorb unto cationic polymers interact with phagocytic cells that have an abundance of receptors on their cell membranes that facilitate significant internalization. When cationic polymers were used in an environment that lacks opsonins, the extent of cell binding and uptake was significantly reduced [49]. Furthermore, uptake of inorganic nanoparticles modified with cationic polymer was considerably increased in SK-BR-3 cell lines compared to nanoparticles with negative charges [50]. It should be noted that positively charged particles may facilitate endosomal escape via the ‘proton sponge effect’ [51], a strategy that circumvents the degradative effect of the acidic and enzyme-rich endo-lysosomal compartment on drug cargo [19].

Strongly negatively charged particles are also rapidly removed by the RES; hence the importance of considering the effect of surface charge during the development of nanoparticles [24]. Research has shown that nanoparticles with neutral and slightly negatively charged surfaces adsorb less plasma opsonins and demonstrate low non-specific cell uptake [52]. To demonstrate this concept, a study evaluated phagocytic and non-phagocytic cell uptake of positively charged rhodamine B (RhB)-labeled carboxymethyl chitosan conjugated nanoparticles (RhB-CMCNP) and negatively charged chitosan hydrochloride conjugated nanoparticles (RhB-CHNP) in various cell lines (L02, SMMC-7721, HEK 293, 786-O, HFL-I or A549 cells). The result showed a high uptake of positively charged nanoparticles (14.8–34.6 mV) by phagocytic cells compared to negatively charged nanoparticles (−13.2 to −38.4 mV). On the flipside, slightly negatively charged nanoparticles were significantly internalized by non-phagocytic cells compared to either positively charged or highly negatively charged nanoparticles [53]. The superior uptake of nanoparticles with small negative charge was likely due to the reduced repulsive forces between the cell membrane and charged species on nanoparticles, as observed from the progressive decrease in cell uptake with increasing negative zeta potential. This result was validated in a biodistribution study of RhB-CMCNP and RhB-CHNP in H-22 tumor bearing mice.

Thus, for effective nanoparticle delivery to tumors, one would desire a neutral or slightly negative nanoparticle surface charge upon intravenous administration, but a switch to positive charge upon arrival at the tumor site [13,19], as demonstrated by the design of a switchable zwitterionic nanoparticle based on TME cues [54]. In this instance, a docetaxel loaded co-polymer was linked to a negatively charged group, i.e., dimethyl maleic acid (DMA), via a pH sensitive amide linker to avoid adsorption of opsonins in plasma. However, at the low pH within the tumor environment, the amide crosslinker was cleaved to release DMA, thereby exposing the positively charged amine groups to enhance adsorptive interaction with the cell membrane.

#### 4.1.3. Shape

The shape of nanoparticles is one of the most researched physicochemical properties (after size) that can be modulated to improve the delivery efficiency of nanocarriers [55]. The shape of nanoparticles has been shown to have remarkable impact on tissue targeting, internalization, immune cell association, cell adhesion, and uptake [56]. The meta-analyses by Wilhelm et al. showed the importance of shape to the overall delivery efficiency of nanoparticles. Their data showed that rod shaped (0.8%) nanoparticles had the highest delivery efficiency compared to spherical shape (0.7%) and others [6]. However, other researchers have explored discoidal shaped nanoparticles because of their unique tumbling and margination dynamics that favor vessel wall interactions considerably more than spherical particles, with implications for better extravasation into the TME [57].

It has also been reported that the shape of nanoparticles is critical to the extent of clearance by macrophages via phagocytosis. This is because geometric parameters, such as curvature and aspect ratio, affect uptake [13]. The kinetics of phagocytosis reveal that particles possessing a length of normalized curvature (designated as Ω) ≤ 45°, as observed with spherical particles, undergo faster internalization than particles with Ω ≥ 45°. The influence of shape on tumor internalization has led to further evaluations of ellipsoidal, cylindrical, and discoidal shaped nanoparticles [13]. The ability of filamentous polymicelles to align with blood flow has been exploited due to their high aspect ratios (>10) and longitudinal length (10μm) that ensures successful retention in the blood for up to a week [53]. This filamentous property enabled the delivery and accumulation of high levels of paclitaxel in tumors when compared to spherical micelles [58].

#### 4.1.4. Elasticity

Elasticity or deformability and biodegradability should also be considered in a bid to enhance EPR. This is because organs like the liver and spleen have fenestrated endothelia that filter rigid particles with diameters that exceed the cut-off limit of their inter-endothelial fenestrae [13]. It has been reported that by decreasing the nanoparticle elastic modulus by eight-fold, the blood circulation half-life thereof can be increased by a factor of thirty [27], hence extending the residence time needed for efficient EPR. In a study that evaluated the influence of elasticity on cellular and tumor accumulation of a PEG-based hydrogel nanoparticle formulation, softer nanoparticles with elastic modulus of 10 kpa demonstrated longer circulation time and enhanced tumor targeting compared to hard nanoparticles with high elastic modulus (3000 kpa). The authors reported a 3.5-fold uptake of hard nanoparticles by macrophages, which explains their short circulation time [59]. Guo et al. [60] investigated the impact of elasticity on cellular uptake of nanoliposomes (NLP), uncrosslinked nanolipogel, and cross-linked nanolipogel (NLG) with varying Young’s elastic moduli (NLP: +45 kpa; and NLG 1.6 ± 0.6 MPa to 19 ± 5 MPa). Their data demonstrate that NLPs and soft NLGs accumulated significantly more in tumors, whereas NLGs with high elastic moduli preferentially accumulated in the liver [60]. Evidence from the work by Hui et al. [61] demonstrated higher uptake of soft silica nanoparticles (560 kPa) compared to hard silica nanoparticles (1.18 GPa) by SKOV3 cell lines, while hard silica nanoparticles were taken up faster by RAW264.7 phagocytic cells [61]. A possible explanation for the increased phagocytosis of hard particles is that very soft particles can potentially undergo deformation in response to the forces of phagocytosis, which can lead to changes in the particle radius of curvature. Deformation that leads to changes such as elongation of nanoparticle shape can reduce susceptibility to phagocytosis. Shape elongation drastically reduces the radius of curvature of nanoparticles, which potentially diminishes the ease of phagocytosis by macrophages [60]. The accumulation of nanoparticles via the EPR effect in these studies seemed to largely depend on the long circulation time of the nanoparticles within the blood.

The impact of deformability on uptake of soft nanoparticles by macrophages may also pose a problem for uptake by non-phagocytic cells. This concern is largely due to evidence from studies that show higher cellular uptake with hard nanoparticles. In one report, hydrogel nanoparticles with intermediate Young’s moduli of 35 and 136 kPa were significantly internalized compared to those with elastic moduli of 18 kPa and 211 kPa [62]. This suggests that an optimum elastic modulus may be essential to improve the EPR effect. The advantages of soft nanoparticles have been contested by other reports. In a recent study on the role of elasticity in the uptake of silicon oxide nanocapsules by HeLa cells, the authors reported that increased elasticity resulted in higher cellular uptake of stiff SiO_2_ nanocapsules by about nine-fold compared to soft nanocapsules [63]. Mechanistic investigation showed that hard nanocapsules were internalized via clathrin mediated endocytosis, while soft nanocapsules were taken up by either the caveolae dependent pathway or via micropinocytosis, as observed in soft nanoparticles with extremely low elastic modulus [60,64].

### 4.2. Enhancing Nanoparticle Navigation in Systemic Circulation

#### 4.2.1. Circumventing Opsonization 

Opsonization is an immune process that involves the adsorption of serum protein fragments (opsonins) to foreign pathogens for recognition and elimination by phagocytes [65]. In the absence of opsonins, the negatively charged cell walls on both pathogens and phagocytes will repel each other, thus enabling the pathogen to replicate uncontrollably while avoiding destruction.

Nanoparticles may attain high concentrations in neoplastic tissues via the EPR effect only if they are able to evade the cells of the reticuloendothelial system [10]. The journey of macromolecules and nanoparticles begins with their injection and continues through different stages of circulation, extravasation, accumulation, endocytosis, endosomal escape, intracellular localization, and pharmacological action [3]. Nanoparticles and macromolecules are particularly prone to opsonization due to their nanosize, that confers a large surface area. The high surface area to volume ratio of nanoparticles generates very high surface energies that engender unusual behavior [48], such as the adsorption of plasma proteins, i.e., serum albumin, apolipoproteins, components of the complement system and immunoglobulins, on the surface of circulating nanoparticles [66]. Opsonization leads to the formation of a protein corona around nanoparticles, a process that is dependent on several factors, such as, nanoparticle size, surface charge, hydrophobicity, and surface chemistry [48]. With the adsorption of proteins, the surface of nanoparticles is primed for attachment to specific receptors on the surface of phagocytes. Phagocytosis then occurs, and the nanoparticles are internalized, transported to phagosomes, and fused with lysosomes [13]. In addition to increasing uptake by the RES, opsonization is detrimental to active-targeting strategies for nanoparticles, since the protein adsorption creates a corona mask that diminishes the binding affinity of targeting ligands, resulting in a marked reduction in specificity [13].

To overcome the challenge of opsonization, hydrophilic polymers that confer stealth properties have been used to drastically reduce the adsorption of opsonins to nanoparticles. The stealth property of nanoparticles implies their ability, via various modification strategies, to navigate biological systems without detection and destruction by the immune system. Modification of the nanoparticle surface to confer stealth property prolongs in vivo circulation time and enhances passive targeting via the EPR effect [67].

The most common strategy to confer stealth property is PEGylation. Using this approach, polyethylene glycol (PEG) is usually grafted, adsorbed, or covalently bonded to the nanoparticle surface [3,13]. Polyethylene glycol is believed to provide a steric barrier, i.e., a hydration zone, around nanocarriers because of its hydrophilicity. This reduces the adsorption of opsonins on the surfaces of nanocarriers, thereby reducing nanoparticle uptake by the cells of the RES in the liver and spleen. This reduced uptake leads to prolonged blood circulation time [68]. The graft density of PEG on the nanoparticle surface is critical to the effectiveness of the PEG coating in resisting protein adsorption. It has been reported that low PEG density leaves regions of the nanoparticle surface exposed to binding by opsonins, while high density may constrain PEG and decimate its ability to push adsorbing proteins away, instead becoming a new surface for protein adhesion [3].

The use of PEG to reduce adsorption of opsonins is not without challenge. Studies [68] have shown that a second dose of PEGylated liposomes in rats or rhesus monkeys was cleared very rapidly from circulation when the interval between the first and second injection was between 5 and 21 days, largely due to enhanced accumulation in the liver. This phenomenon, commonly referred to as ‘accelerated blood clearance’, is responsible for the sudden decrease in nanocarrier concentration after subsequent injections of PEGylated nanoparticles and macromolecules. This leads to short circulation time and low accumulation of nanoparticles at tumor sites, thus compromising efficacy. The rational design of nanoparticles requires that more attention be paid to the ABC effect and its overall impact on EPR.

To avoid PEG-induced ABC effect, various hydrophilic synthetic coatings (polyvinylpyrrolidone, PVP; polyphosphoesters, PPEs; polyelectrolytes, and zwitterionic polymers) and natural polymeric coatings (polynucleotides, polypeptides, dextran, and chitosan) have been explored with the goal of preserving the physicochemical properties, surface properties, and functional integrity of nanoparticles within biological systems, to prolong blood circulation. These agents mitigate ABC by preventing protein corona formation, warding off RES cells, and averting nanoparticle agglomeration, as well as preventing other bio-nanoparticle interactions that serve as barriers for effective nanoparticle drug delivery [69]. Another strategy involves the use of polyglycerol (PG) lipids for modification of nanocarrier surfaces. Application of PG to a liposome nanocarrier was shown to produce neither an anti-polymer immune response nor the ABC phenomenon upon repeated administration, resulting in enhanced therapeutic efficacy of encapsulated doxorubicin in a tumor-bearing mouse model [68]. Similarly, a novel cleavable PEG lipid derivative (mPEG-Hz-CHEMS) in which the PEG moiety was linked to cholesterol by two ester bonds and one pH-sensitive hydrazone molecule has been reported as a promising PEG alternative [70]. The authors revealed that liposomes functionalized with this novel PEG-lipid derivative demonstrated prolonged blood circulation characteristics, and upon repeated administration, showed no ABC phenomenon compared with liposomes modified with mPEG-CHEMS lipid derivative.

Hydrophilic synthetic materials such as zwitterionic polymers have received a lot of attention in recent years. Zwitterionic polymers are made of moieties with both cationic and anionic groups, characterized by high dipole moments from highly charged species, and yet maintain charge neutrality [71]. Zwitterionic materials are biocompatible, resist nonspecific protein adsorption in the blood, and do not induce immunological response in vivo [72]. The mechanism by which zwitterionic materials prevent nonspecific protein adsorption involves strong electrostatic interactions between highly charged groups that induce hydration around nanoparticles, thus preventing biofouling. Although both PEG and zwitterionic polymers induce hydration, besides the non-induction of immunologic responses, zwitterionic materials differ in their structure and extent of hydration. They have been shown in molecular dynamics simulations to possess lower free energy of hydration, which translates to stronger hydration. Lower free energy of hydration leads to low water mobility and wider dipole moments that together repulse adsorption of proteins and other charged species, respectively [71].

Zwitterionic materials are made from small molecule zwitterions like phospholipids, betaine, amino acids, and their derivatives. Polymeric zwitterions are formed from the surface derivatization of nanoparticles, proteins, and hydrogels, and include polycarboxybetain acrylamide (polyCBAA), polycarboxybetaine methacrylate (polyCBMA), and polysulfobetaine methacrylate (polySBMA) [73]. The efficiency of zwitterion polymers in enhancing drug delivery and EPR has been demonstrated in several studies. In one study, the ability of a uricase-loaded pCB nanocarrier to prevent humoral immune response (from either uricase or polymer) and reduction in efficacy after repeated administration was investigated. The result showed that in a clinical rat model of gout, repeated administration of pCB loaded carrier was superior to pegylated uricase, as indexed from a lack of immune response and sustained efficacy [74]. In a different study, the biocompatibility and circulation time of a pCB-based nanocarrier were comparable to those of a pegylated system [75]. The conjugation of polycarboxybetain polymers to organophosphate hydrolase increased its pulmonary delivery due to a significant increase in bioavailability (5% to 53%) [76]. The co-conjugation of docetaxel and curcumin onto a polycarboxybetaine (pCB) polymer led to a significantly enhanced EPR effect in multidrug resistant MCF-7/Adr cell lines due to the antifouling effect of pCB. The result showed cell cytotoxicity at IC_50_ of 5.87 μg/mL for docetaxel-curcumin-pCB, compared to either docetaxel (437.2 μg/mL) or pCB-Dox (14.1 μg/mL) [77]. Delivery of docetaxel via EPR effect in a zwitterionic shielded, pH-responsive folate conjugated polymer has also been reported [78]. In this construct, a zwitterionic co-polymer was synthesized via reversible fragmentation chain transfer (RAFT) copolymerization from 2-(methacryloyloxy)ethyl phosphorylcholine and polyethylene glycol methacrylate ester benzaldehyde. The authors reported that upon conjugation of the docetaxel-loaded zwitterionic co-polymer to folate and exposure to HeLa cells, there was rapid and efficient internalization due to the presence of folate, and strong interaction between multivalent phosphorylcholine (PC) groups and cell membranes. Such efficient uptake has potential to enhance the EPR effect in vivo.

#### 4.2.2. Overcoming the Impact of the Reticuloendothelial System 

The reticuloendothelial system (RES) is a diverse collection of phagocytic cells expressed in systemic tissues as part of the innate immune system that are actively involved in the elimination of particles and soluble substances in both blood circulation and tissues. The RES consists broadly of Kupffer cells of the liver, microglia of the brain, alveolar macrophages, bone marrow, lymph nodes, and macrophages of the intestine and other tissues [79]. Typically, less than 5% of an injected dose of nanoparticles is delivered to the cancer tissue, resulting in an extremely low drug delivery efficiency and, consequently, a poor therapeutic outcome [80]. Elimination of the bulk of nanoparticles occurs via the RES organs of the liver and spleen [81]. The injection of large amounts of drug-loaded nanoparticles to compensate for this loss raises toxicity concerns with respect to decreased RES function and the risk of nonspecific drug release [80]. Therefore, reducing the uptake of nanoparticles by the RES is a strategic approach to enhance the magnitude of the EPR effect that could lead to high accumulation of macromolecules at the site of action.

Prominent among the RES apparatus are the Kupffer cells positioned in liver sinusoids as part of the body’s innate immunity [82]. They are specialized macrophages formed from liver adhering circulating monocytes that polarize into cells with highly differentiated surface receptors which facilitate the binding and/or uptake of foreign materials [27]. The extent of nanoparticle uptake and retention in Kupffer cells is strongly associated with the nanoparticle’s surface charge, ligand chemistry, and size, with particles of highly cationic and anionic surface charge being cleared faster than those with neutral charge [83]. Similarly, large nanoparticles are more likely to be sequestered and destroyed by the Kupffer cells [20]. The fate of nanoparticles is further complicated by the fact that smaller monodispersed nanomaterials may be taken up by liver sinusoidal epithelial cells (LSECs) to a higher degree [27]. This suggests that escape from the Kupffer cells does not imply freedom to circulate. Hence, the importance of the rational design and optimization of the physicochemical properties of nanoparticles should be obvious.

##### Enhancing Nanoparticle Delivery by Silencing or Depleting Kupffer Cells

A strategy to improve the EPR effect is to silence the Kupffer cells, since they are responsible for the bulk of nanoparticle sequestration. This approach is based on the principle that when liposome loaded with clodronate or other agents are taken up by macrophages, the phospholipid bilayers of liposomes are digested by lysosomal phospholipases to release clodronate, which inhibits ADP/ATP translocase in the mitochondria and ultimately triggers the apoptosis of macrophages [23]. The advantage of this strategy is that only phagocytic cells are targeted, and the remaining clodronate are eliminated via the renal system, leading to an extremely short half-life in the bloodstream. Nevertheless, the drawback of this strategy is that at high doses, the depletion of splenic macrophages may result in splenomegaly that may predispose patients to sepsis [84].

Tavares et al. [85] were able to increase nanoparticle delivery to tumor 150-fold by removing all or a portion of Kupffer cells. They reported a series of experiments that involved dose-dependent reduction of macrophages using dichloromethylene diphosphonic acid liposomes (clodronate liposomes), followed by i.v. administration of various nanoparticles (gold-, silver-, silica nanoparticles, and liposomes) in different xenograft models (ovarian, breast, skin, prostate, and lung cancer) in a two-step dosing schedule spread over 48 h. Their data showed that tumor accumulation of nanoparticles increased 150-fold for 50-nm gold nanoparticles, while there was 100-fold increase in tumor accumulation in animals with PC3 orthotopic xenografts for 100-nm gold nanoparticles 48 h after i.v. administration of clodronate liposomes. The data showed that although there were high blood levels of nanoparticles with almost 98% bioavailability, only 2% was delivered to the tumor site.

Minimizing the sequestration of nanoparticles by Kupffer cells is expected to dramatically increase tumor accumulation of nanoparticles. The low tumor accumulation in a situation of diminished Kupffer cell activity begs the question of the contribution of other organs such as the skin, lymph nodes, spleen, and lungs, as well as the pathophysiology of the tumor to nanoparticle sequestration and drug delivery [85]. Tavares et al. also evaluated the possibility of infection during periods of Kupffer cell depletion by using a polymicrobial model of sepsis to determine the outcome of infection in a depleted Kupffer cell situation. The result showed that for acute infection during the period of Kupffer cell depletion therapy, the prognosis will be less favorable. In addition, the study by Tavares et al. revealed that tumor accumulation of nanoparticles, although low, is largely dependent on nanoparticle size, material composition, and tumor type. Thus, optimizing the physicochemical properties of nanoparticles is a rational and strategic initial step towards enhancing the EPR effect.

Other research groups have used gadolinium chloride (GdCl_3_), a Kupffer cell deactivator, to inhibit the function of Kupffer cells by suppressing phagocytosis via inhibition of calcium transport across the cell membrane [86,87,88,89]. In one such example, it was demonstrated that a significant number of liver Kupffer cells were inactivated after pretreatment with systemic administration of GdCl_3_, leading to an increase in the circulatory half-life of Quantum dots, and consequently, a 50% increase in tumor-specific uptake [87]. Clodronate and gadolinium chloride have no effect on liver sinusoidal epithelial cell uptake and may be more beneficial to inhibiting liver sequestration of larger nanomaterials. While attractive, these transient depletion strategies are not well-characterized for their safety, and studies investigating dose–efficacy relationships and concurrent effect on innate immunity are rare [23].

Another research group proposed the use of intralipid 20%, an FDA-approved fat emulsion used for parenteral nutrition to temporarily blunt the phagocytic capacity of Kupffer cells by decreasing the accumulation of nanoparticles in the liver and spleen, thus increasing the bioavailability of nanodrugs via the EPR effect [90]. This strategy stems from a report that infusion of Intralipid 20% impedes Kupffer cell function by inhibiting peritoneal clearance, hence impairing their phagocytic activity [91]. Their data showed that in rodents, intralipid reduced Kupffer cell uptake by approximately 50%, leading to an increase in the blood half-life of nano- and micron-sized super paramagnetic iron-oxide particles by ~three-fold. They also demonstrated that a single clinical dose (2 g/kg) of intralipid 20% could decrease the accumulation of platinum nanoparticles in the liver by 20.4%, in the spleen by 42.5%, and in the kidney by 39.3% after 24 h post nanodrug administration. Subsequently, the bioavailability of the platinum-nanodrug increased by 18.7% during the first 5 h and by 9.4% after 24 h, respectively.

Most strategies aimed at silencing the impact of the RES have focused solely on Kupffer cells. Earlier, it had been established that small-sized nanoparticles are more likely to be trapped within the liver sinusoidal endothelial cells (LSEC), which could affect the overall bioavailability of these particles. The major receptors involved in the sequestering action of liver sinusoidal epithelial cells include mannose, Fcγ, collagen-alpha receptor, and the hyaluronan scavenger receptors [27]. Kupffer cells equally express mannose and Fcγ receptors. These two receptors could be targeted in both Kupffer cells and LSECs by pretreating with a combination of different sized nanoparticles bearing inhibitors of both cell types, designed to fit the size threshold of Kupffer cells (≥100 nm) and LSECs (<30 nm).

##### Enhancing Nanoparticle Delivery by Saturating Kupffer Cells

The saturation of the receptors of Kupffer cells with bait and nontoxic unloaded nanoparticles prior to dosing of nanotherapeutics may enhance nanoparticle accumulation in neoplastic tissues [27]. In one study, liposomes made of phosphatidylcholine and cholesterol were used to saturate phagocytosis by macrophages [91]. The subsequent inhibition of phagocytosis occurred within 90 minutes after dosing with liposomes, resulting in an increased intratumoral accumulation that persisted for 48 h. The application of this strategy led to a two-fold increase in the accumulation of PEGylated nanoparticles in a human prostate cancer xenograft model after a single dose, compared to controls [91]. Unlike the Kupffer cell-depleting strategy mentioned in the previous section, the Kupffer cell saturation approach is safe and does not damage the innate immunity, as reflected in the lack of weight loss, non-impairment of liver function, and unchanged host defense in the experimental animals used [91]. It should be noted, however, that this strategy is limited by the fact that the phagocytic function of Kupffer cells was not fully inhibited in this study, judging from the impact of only a two-fold improvement in tumor accumulation. Further development may require the use of nanoparticles made of materials that have slower degradation rates [27], or the titration of the blank decoy nanoparticles to evaluate the kinetics of optimum accumulation.

A technique which overwhelms the uptake rate of nanoparticles by Kupffer cells resulting in decreased hepatic clearance has been used to establish high tumor accumulation of nanoparticles. In a study which focused on evaluating the relationship between nanoparticle dose and liver clearance [92], it was hypothesized that the proportion of nanoparticles taken up by the Kupffer cells in the liver would decrease considerably if the dose were increased beyond the uptake rate of Kupffer cells. The aim of this strategy was to find a threshold nanoparticle dose that would minimize liver clearance without compromising liver function. In line with the desire to translate preclinical findings to the clinic, the authors sought to describe the administered dose in terms of an equivalent number of nanoparticles rather than the dose derived from pharmacological allometry. To determine the relative number of nanoparticles required to saturate Kupffer cells, in vivo experiments [92,93] revealed an estimate of 10 million Kupffer cells in the mouse liver and a projected total clearance rate limit of about 1 trillion nanoparticles per 24 h. Therefore, a gradual increase in the dose of nanoparticles above 1 trillion particles is expected to result in a progressive saturation and decrease in Kupffer cell activity. Liver mass extrapolation estimates 8, 63, and 1.5 quadrillion nanoparticles for rat, rabbit, and a 70-kg man whose livers weigh 8, 63, and 1500 times that of the mouse, respectively. At nanoparticle doses below these thresholds, rapid liver elimination would occur, leading to suboptimal accumulation in the tumor [92].

**Figure 2:**
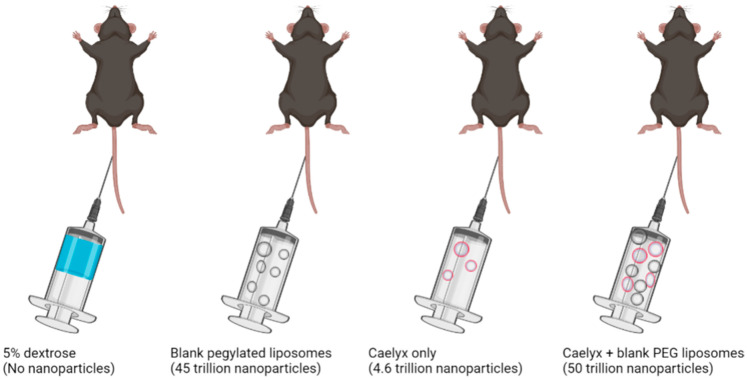
Scaled dosing of nanoparticles according to threshold required to overwhelm Kupffer cells. Adapted from [92].

To prove this hypothesis, Ouyang et al. [92] intravenously injected 4T1 tumor-bearing BALB/c mice (mammary carcinoma cell mouse model) with variable quantities of 50 nm-sized polyethylene glycol-conjugated (PEGylated) gold nanoparticles (ranging from 50 billion to 50 trillion nanoparticles). Their data showed that the liver sequestered less pegylated gold nanoparticles with increasing dose, leading to an increase in the blood half-life from 2 min to 8 h. This strategy was repeated by the same authors in a proof-of-concept study by pretreating xenograft mice models with blank pegylated liposomes (Figure 2) to overwhelm the uptake rate of Kupffer cells before administering Caelyx^®^ (PEGylated doxorubicin loaded liposomes). The result showed a 12% accumulation of injected dose at the tumor site without any record of death, despite the known toxicity of doxorubicin. The reader is referred to the study by Ouyang et al. for a detailed description of their technique.

To reiterate the importance of optimal dosing in overcoming nanoparticle sequestration and improved tumor accumulation, it has been reported that investigational agents, i.e., BIND-014 and NK105, that failed in clinical trials, were dosed below the 1.5 quadrillion threshold, at 1.0 and 0.9 quadrillion nanoparticles per patient, respectively [93], despite being optimized for size, ligand density, drug encapsulation, and release kinetics [94]. Possibly, high sequestration by Kupffer cells and suboptimal delivery to tumors may have been responsible for their therapeutic failure.

### 4.3. Promoting Nanoparticle Delivery with Circulating Cells

Despite improvements seen with the implementation of passive- and active targeting techniques to increase delivery and tumor accumulation of macromolecules, a large percentage of these particles are still cleared by the RES, with a small fraction reaching the tumor microenvironment, thus limiting the clinical translation of nanoformulations. The development of strategies to evade the RES is therefore a key element in fashioning delivery systems that can remain in the blood circulation for long periods to facilitate the efficient accumulation of nanoparticles within solid tumors by the EPR effect [23]. To achieve improved accumulation, several approaches have been proposed, such as ‘back packing’, ‘cellular hitchhiking’, and ‘Trojan Horse’ strategies, that exploit the natural ability of cells within the circulatory system to evade the immune system and be transported via natural tropism to specific, vascular, or systemic locations around the body while crossing biological barriers that are otherwise nearly impermeable [95]. Hence, modeling nanocarrier designs from nature or other bioinspired approaches have been evaluated for reducing nanoparticle uptake by the cells of the RES in the liver [27]. These techniques use, *inter alia*, circulating red blood cells, leukocytes, and monocytes that differentiate into macrophages.

#### 4.3.1. Nanodelivery with Red Blood Cells (RBCs)

**Figure 3:**
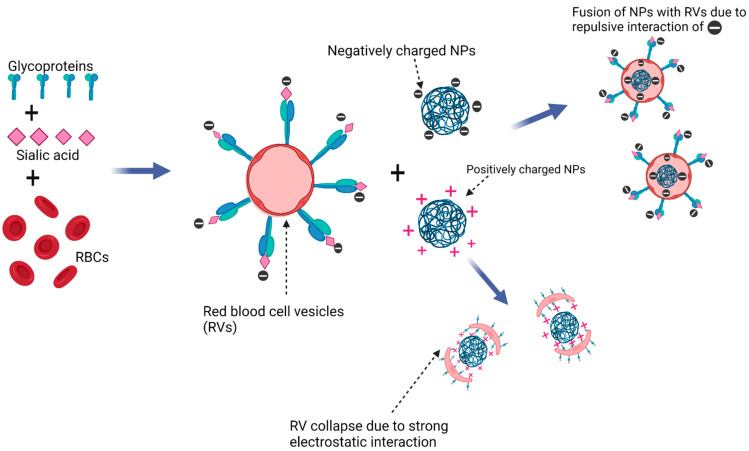
Preparation of RBC vesicle-coated nanoparticles. Schema of electrostatic interactions between negatively and asymmetrically charged RVs (red blood cell vesicles) with negatively charged nanoparticles on one side and positively charged ones on the other. First, the RBCs are designed to form negatively charged vesicles by the addition of glycoproteins and sialic acid. Secondly, the RVs are then mixed with positively and negatively charged nanoparticles, respectively, with their embedded payloads. Naturally, the negatively charged nanoparticles strongly repulsed the negatively charged RVs; however, fusion was achieved by co-extrusion, where mechanical force drives the nanoparticles through the lipid bilayer to fuse with the intracellular membrane side of RBCs. The strong affinity between the positively charged nanoparticles and the negatively charged RVs led to the collapse of the lipid bilayer, which prevented nanoencapsulation. Modified from (Ref. [98]) and Created with BioRender.com Accessed on 24 October 2021.

Red blood cells (RBCs) are of particular interest due to their safety, abundance, and life span of approximately 120 days [96]. A group of researchers evaluated the use of organic nanoparticles as RBC hitchhikers by developing a smart RBC system containing doxorubicin and bovine serum albumin nanocomplexes for the chemo- and photothermal therapy of glioblastoma cells [95]. These RBCs were further functionalized with RGD peptide (arginine-glycine-aspartic acid) to target the integrins of the endothelium of tumor blood supply. The challenge with using RBCs is that they lack phagocytotic properties (making it difficult to incorporate nanoparticles into them), and they are prone to membrane disturbances especially during processing (adsorption, electrostatic or covalent interactions) with therapeutic agents or carrier molecules, leading to agglutination, stiffness, increased sensitivity to osmosis, and mechanical and oxidative stress that increases the chances of exposure of membrane phosphatidylserine [97]. Research has shown that exposure of phophatidylserine and other ‘eat me’ signals are responsible for the recognition and phagocytosis of perturbed and dying cells by the RES [97]. By applying the Trojan Horse strategy, a research group successfully loaded nanoparticles into RBC vesicles via a co-extrusion method. This was done by decorating the surface of RBC vesicles with negatively charged sialyl residues via polysaccharide linkers to confer charge asymmetry on the RBC membrane. This charge asymmetry facilitated the fusion of nanoparticles with RBC vesicles when subjected to co-extrusion [98] (Figure 3).

#### 4.3.2. Nanodelivery with Monocytes and Macrophages

Leukocytes (granulocytes, monocytes and, lymphocytes) are found in large quantities in the blood (4−10 billion) and have circulation times of about three weeks [95]. They constitute the body’s adaptive and innate immunity, and are widely investigated as nanoparticle vehicles due to their natural tropism that allows movement through endothelial barriers to sites of disease and hypoxia, like the TME [23,99]. Monocytes are produced in the bone marrow and differentiate into macrophages in deep tissues and organs, where they detect and phagocytose necrotic cells, pathogens, and sundry foreign particles through their specialized membrane receptors [23]. Macrophages can also circulate to inflammatory and hypoxic regions, as well as cross the blood−brain barrier via diapedesis and chemotaxis, thus making them a versatile ally in the delivery of nanoparticles [99].

Notwithstanding the promise of efficient nanoparticle delivery by macrophages, the loading of large quantities of nanoparticles into macrophages remains a challenge. A typical strategy employed in loading nanoparticles into macrophages is the ‘backpack’ approach, which involves conjugating drug molecules or drug-loaded nanoparticles onto the plasma membrane [100]. This “backpack” approach has been used to load therapeutic nanoparticles onto stem cells, leukocytes, red blood cells, and T cells, with varying degrees of success [96,100]. In this approach, however, since the plasma membrane is essential for cell function and plasticity, conjugated nanoparticles may adversely affect cell signal transduction, adhesion, and migration. In addition, the number of nanoparticles that can be loaded on the membrane at a time is limited. Furthermore, monocytes and macrophages can readily engulf the nanoparticles loaded on the surface of their plasma membrane, thus diminishing the overall efficiency of the strategy [100].

The alternative strategy of loading drugs into the cell cytosol (‘Trojan Horse strategy’) is also difficult, because most anticancer drugs are highly toxic to macrophages, and hence, the encapsulation of high concentrations of drugs in macrophages could induce immediate cell death, while the encapsulation of low drug concentrations may lead to insufficient drug loading and sub-lethal dosing [100].

The direct conjugation of nano-constructs to the surface of macrophages and the Trojan Horse strategy are usually performed ex vivo, starting with the harvesting of plasma, followed by surface modification or nanoencapsulation, before reintroducing the modified macrophages back to the animal. In a bid to resolve the challenges associated with loading macrophages with nanoparticles ex vivo, a research group developed drug-silica nanocomplexes (DSN-Mf) with negative charges to increase interaction with loaded drugs, reduce the propensity of drug release and thereby decrease the possibility of death of carrier macrophages [100]. The silica nanoparticle was designed in a manner that retarded the release of the drug payload over a period of 48 h. The incubation of macrophages with nanoparticles was limited to 2 h for optimum nanoparticle loading without overwhelming the macrophages. In a U87MG subcutaneous tumor xenograft, the macrophage-loaded silica nanoparticles showed an impressive tumor growth inhibition rate of 62.66% on day 14, with a substantial extension of the animal median survival to 26 days, compared to 14 days in the control group.

In a different study, doxorubicin-containing echogenic liposomes were loaded into polycation (polyallylamine hydrochloride (PAH), polydiallyldimethylammonium chloride (PDAC)) and polyanion (polyacrylic acid (PAA) or polystyrene sulfonate (SPS)) films in a layer- by-layer assembly before ‘back packing’ to the surface of monocytes [101]. Silicon wafers were used as substrates to create a polyelectrolyte multilayer film by incubating it with one polycation (PAH or PDAC) and one polyanion (PAA or SPS) layer. The polycation was used as the first and last layer to increase the number of positive groups on the film surface to engender firm interaction with liposomes that are naturally electronegative. This multilayered film was then mixed with liposomes, followed by a second layer of multilayered film to create a vesicle of liposome-loaded doxorubicin sandwiched between both layers. The multilayered liposomal construct was then backpacked to the surface of mouse monocytes to evaluate its efficacy. The result showed that use of echogenic liposomes for drug encapsulation into backpacks enabled up to three times DOX loading compared to backpacks without echogenic liposomes. In vitro cytotoxicity evaluation revealed that monocyte backpack conjugates remain viable even after 72 h, demonstrating their promise as a drug delivery vehicle.

#### 4.3.3. Cellular Hitchhiking with Macrophages

Hitchhiking involves the use of live macrophages in vivo without modifying the membrane. The previously discussed methods (Section 4.3.2) of conjugating and encapsulating macrophages are expensive and limited in the amount of drug that can be loaded. However, by targeting circulating monocytes, its natural phagocytic properties can be harnessed for nanoparticle loading, with the idea that upon homing to the tumor site, they can differentiate into macrophages and serve as “Trojan Horses” to release their cargo deep within the hypoxic tumor in a controlled manner [23]. For example, Yang et al. [102] used live circulating monocytes to hitchhike a docetaxel-loaded polymeric-micelle formulation synthesized from chitosan and stearic acid. The authors chose chitosan for its biocompatible and biodegradable properties and stearic acid for its cell membrane compatibility that promotes cell uptake. To enhance uptake by monocytes, the micelle batch with particle size of 86 nm and positive zeta potential (23 mv) was chosen for further investigation. This is because positive zeta potential values promote adsorption of opsonins which, in turn, promotes uptake by circulating monocytes. Tumor delivery was achieved by exocytosis of the drug loaded micelle upon differentiation of monocytes to macrophages at the tumor site [102].

In an innovative strategy, Zhang et al. [103] exploited circulating macrophages to target CD47-rich tumor cells in a 4T1 murine breast cancer model. These authors designed drug-loaded silicon nanoparticles and decorated the surface with calreticulin and anti-CD4 antibody to enhance the EPR effect. The mechanism of this strategy is to diminish the ‘don’t eat me’ signal of CD47-rich tumor cells with anti-CD47 antibodies while promoting ‘eat me signal’ with calreticulin. The findings show that the simultaneous application of anti-phagocytic and pro-phagocytic signals can significantly enhance macrophage-mediated cancer delivery [103]. The reader is referred to the review by Izci et al. [23] for a detailed description of other hitchhiking strategies with circulating monocytes and macrophages.

#### 4.3.4. Enhancing Drug Delivery with Other Cell-Based Strategies

Platelets are circulating cells that are devoid of a nucleus but are useful as delivery vehicles because of their sensitivity toward inflamed tissues upon activation. Such sensitivity can be harnessed for the delivery of therapeutic payloads to platelet-activating tumors [101,104]. In one report, to overcome cancer recurrence after surgical resection, anti-PDL-1 antibodies were conjugated to the cell surface of mice-derived platelets via a maleimide linker for delivery to the freshly resectioned sites. The administration of platelet bound anti-PDL1 considerably prolonged overall mouse survival after surgery by reducing the risk of cancer regrowth and metastatic spread [105].

Extracellular vesicles (EVs) derived from mesenchymal stem cells (MSC) have been used for nanodelivery to improve the EPR effect [106]. EVs are lipid bilayer membrane vesicles that are produced by eukaryotic cells from several regulatory processes involving endocytosis, fusion, and efflux. They are less immunogenic, relatively non-toxic, and can penetrate tumors and inflamed tissues [107]. The RES clearance threshold for EVs is relatively high, making them useful for stealth delivery. Wei et al. [107] exploited this attribute to deliver microRNA (miR21)-loaded CD47-EVs to inflammatory cardiac cells in a mouse model of acute myocardial infarction and ischemia-reperfusion. The result showed longer circulation time (120 vs. 30 min) and preferential accumulation of CD47-EVs in inflamed cardiac tissues compared to unmodified EVs.

Cancer cell membranes are known to specifically recognize homologous cells, persist for a long time in blood circulation, and possess the capacity to evade phagocytic cells. As such, they are useful in the formulation of nanocarriers for improving the EPR effect [108]. Fang et al. [109] demonstrated the EPR enhancing effect of cancer cell membranes for drug delivery in a construct of PLGA nanoparticles coated with B16−F10 mouse melanoma cells without intracellular content. The data showed that when exposed to MDA-MB-243 cell line, cancer cell membrane-coated nanoparticles (CCNP) were significantly internalized compared to either RBC coated PLGA or plain PLGA. In addition, the CCNP induced a cancer-directed immune response [108]. This construct may be further optimized by encapsulating antineoplastic agents within the PLGA core for dual effect. Chen et al. [110] present evidence of enhanced EPR effect with an indocyanine green-loaded and cancer cell membrane-coated PLGA-PEG nanoparticle. The cancer cell membrane was derived from MCF-7 cancer cells. The study show that biomimetic nanoparticles significantly promote cell endocytosis and homologous-targeting of MCF-7 tumor xenograft, leading to substantial accumulation in vivo when indexed against plain indocyanine green and PLGA. Moreover, the biomimetic nanoparticles persisted in circulation for a long time at 7–14-fold concentration of both plain indocyanine green and PLGA due to reduced sequestration and elimination by the liver and kidney [110].

### 4.4. Enhancing Nanodelivery via the “Don’t Eat Me Strategy”

**Figure 4:**
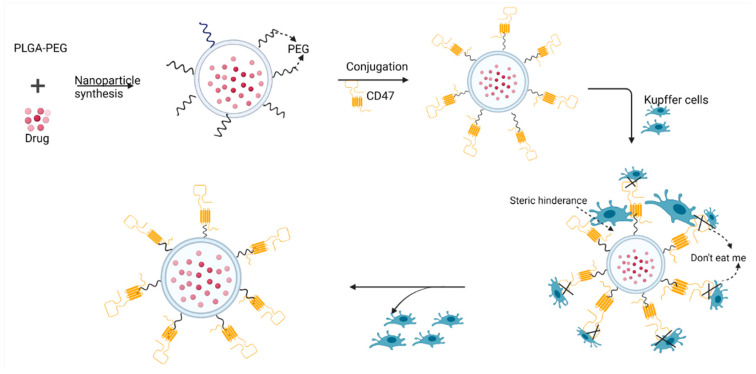
Preparation of a PLGA-PEG nanoparticle loaded with drug molecules and coated with CD47 peptides to prevent phagocytosis by Kupffer cells. Created with BioRender.com Accessed on 30 January 2022.

Another evasive technique to avoid cells of the RES is the ‘active stealth’ approach explored by Rodriguez and coworkers [111]. In their experiment, CD47 (a putative self-marker) was used to decorate the surface of nanoparticles, making them biomimetic and recognizable by the macrophages of the blood and Kupffer cells as ‘self’, thus avoiding phagocytic clearance (Figure 4). In this study, CD47 ‘self’ peptides were computationally designed, synthesized, and attached to 160-nm paclitaxel-encapsulated nanobeads, followed by administration to NSG- (NOD) severe combined immunodeficient *IL2rγ null* mice. The self-peptide (CD47) functionalized nanobeads substantially prolonged nanoparticle circulation by impairing phagocytic clearance by the RES. An in vivo evaluation of nanoparticles derivatized with the CD47 ‘self-peptide’ showed superior accumulation in A549 tumors within 10 min of administration, followed by release of the encapsulated paclitaxel, resulting in significant tumor shrinkage compared to the conventional Cremophor EL formulation of the drug [111].

To increase tumor accumulation, nanoparticles derivatized with CD47 peptides may also be decorated with targeting moieties that e home in specifically on exclusive targets within the TME. Alternatively, CD47 peptides may be conjugated to nanoparticles via a cleavable pH labile hydrazone bond that can only be cleaved in a highly hypoxic environment, thus ensuring exclusive delivery while preventing non-specific distribution [68,112,113].

### 4.5. The Synthetic Microbe Strategy

Intracellular pathogens have evolved with unique instruments to evade the immune system and thrive within host cells, making them difficult to treat [114]. An understanding of these microbial evasive mechanisms provides an opportunity to apply such survival strategies for drug delivery. The repurposing of bacterial effectors in an appropriate combination for the development of a macrophage-based delivery system for the conveyance and controlled delivery of therapeutic agents packaged in a “synthetic microbe” has been proposed [114].

**Figure 5:**
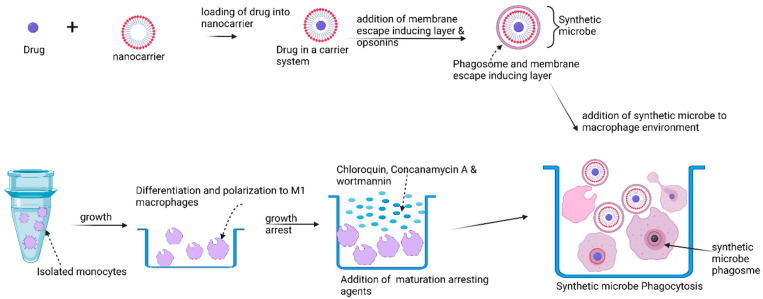
Formulation of the proposed synthetic microbe. Drugs are first loaded into an appropriate nanocarrier and coated with a membrane escape-inducing layer, similar to macrophage escape effector proteins in micro-organisms. The membrane-inducing layer is further coated with opsonins (a phagosome-inducing layer) to facilitate uptake by macrophages. The next step involves monocyte isolation and differentiation into M1 macrophages. They will be left to grow, but maturation will be arrested by the addition of chloroquine, concanamycin, and wortmannin. Maturation arrest is required to prevent the premature release of the synthetic microbes from the macrophages in vivo. The last step is the introduction of the synthetic microbe in the microphage environment to activate phagocytosis. Adapted from (Ref. [114]) and Created with BioRender.com Accessed on 30 March 2022.

**Figure 6:**
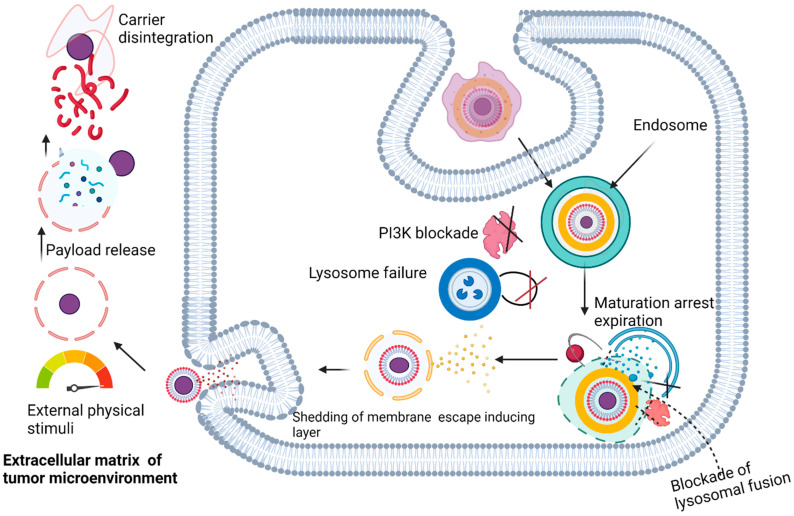
Mechanism of drug release from synthetic microbe. The microbe undergoes endocytosis but is not degraded due to the presence of effectors that block the attachment of PIK3 and lysosomes. Upon expiration of the macrophage maturation arrest, the macrophage degrades the membrane escape-inducing layer (effectors), thereby releasing the drug-containing nanoparticles, which expel the drug upon application of an external physical stimuli at the tumor site. Modified from Ref. [114].

In this proposed scheme (Figure 5), the drug is first incorporated into two layers of nonbiodegradable but biocompatible nanoparticles with built-in membrane-escaping agents (LLO, pore forming listeriolysin-O, actin inducing protein (ActA), and actin polymerizing complex (ARP2/3) that mimic microbial effectors such as those found in *Listeria monocytogenes*. The microbial effectors in *Listeria monocytogenes* allow the expulsion of mature *Listeria monocytogenes* cells without macrophage destruction. The surface of this drug-loaded construct is decorated with opsonins to promote phagocytosis, and then incubated with macrophages together with wortmannin, chloroquine, and concanamycin to arrest phagosome maturation, thus preventing intracellular degradation. Macrophage encapsulation of the synthetic microbe is meant to proceed for a maximum of 1 h to avoid overloading the macrophages. The loaded macrophages are thereafter injected into experimental models for in vivo evaluation. It is expected that natural tropism and chemotactic mobility will drive the migration of the synthetic microbe to tumor cells. Final drug release will be dependent on the built-in release mechanism. For instance, if temperature sensitive poly-N-isopropylacrylamide nanoparticles are used, then an increase in temperature can be used to trigger release. Likewise, ultrasound may be used to trigger release if microbubbles are used (Figure 6). In terms of safety, drugs may be released prematurely from macrophages but are less likely to be expelled from the nanoparticles, except at the site of external delivery support.

### 4.6. Exploiting Glutathione-Mediated Biotransformation

Glutathione efflux from hepatocytes into liver sinusoidal endothelial cells mediates the biotransformation of small foreign molecules, leading to their elimination from the body through the renal or hepatobiliary system [115]. Although the sequestration and clearance of nanoparticles from systemic circulation by cells of the RES has been well studied [27], the mechanism of glutathione biotransformation remains unclear. A recent study [115] reported prolonged blood circulation time of gold nanoparticles with a substantial improvement in EPR effect in a mouse model of human breast cancer (MCF-7) mediated by a glutathione biotransformation mechanism. In this study, glutathione-coated gold (Au25) nanoclusters (GS-Au25) were conjugated to indocyanine green (ICG_4_) to promote protein adhesion (opsonization), thus ensuring delivery of the nano-construct to the liver for biotransformation. Opsonization also increases the hydrodynamic size of GS-Au25 and prevents renal elimination of the fraction that goes straight to the kidney.

**Figure 7:**
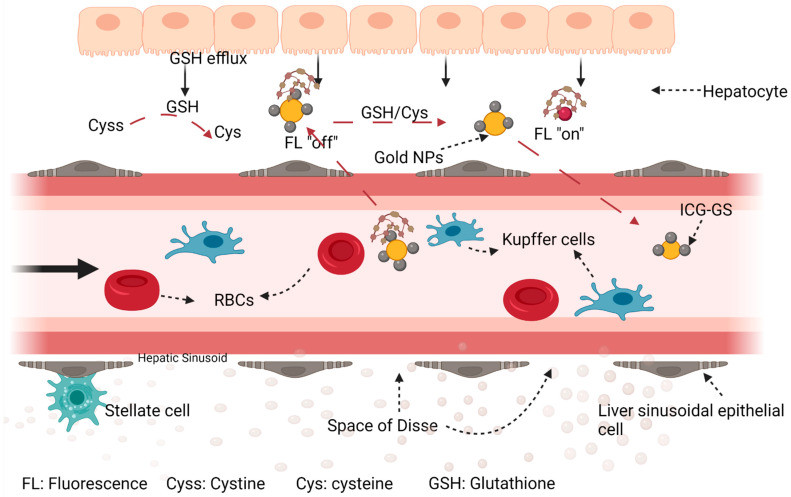
GSH secreted from hepatocytes converts extracellular cystine (Cyss) to cysteine (Cys), which together with GSH, undergo disulfide bond interaction with ICG_4_-GS-Au25 conjugates thereby displacing protein bound ICG-GS from the surface of gold nanoparticles (Au25). The displaced ICG-GS (whose fluorescence was suppressed while bound to gold nanoparticles) regains fluorescence and become susceptible to renal clearance. After periods of residence within the body, enough time for a chance to extravasate into the tumor microenvironment, untransformed ICG_4_-GS-Au25 is eventually broken down and eliminated. Modified from (Ref. [115]) and created with BioRender.com Accessed on 15 April 2022.

The small size of the nano-construct ensures that despite an increase in the hydrodynamic size due to opsonization, the overall diameter remains below the kupffer cell phagocytic threshold thus escaping sequestration and migrating preferentially to liver sinusoidal epithelial cells where it engages in disulfide exchange with the local high concentration of glutathione and cysteine. Upon biotransformation, some, or all the ICG_4_ on the surface of Au25 may be shed to reduce the protein-binding affinity on Au25 (Figure 7). The ICG-GS dislocated as part of the disulfide exchange reaction is taken up by hepatocytes and eliminated through the hepatobiliary pathway while the transformed Au25 nanocarrier goes into circulation for tumor targeting together with the untransformed ICG_4_-GS-Au25 via the EPR effect. The outcome of this strategy showed that tumor targeting improved 27-fold after 24 h compared to the ICG control while the EPR effect of the ICG-conjugated construct was 2.3 times higher than the non-conjugated control.

This strategy can be applied to nanoparticles between 5 nm–100 nm if they are designed to interact efficiently with liver sinusoidal GSH. This assumes that biotransformation occurs side by side with the actions of the RES and a design that ensures rapid biotransformation will reduce phagocytic uptake by the RES. Tumor accumulation can be further improved with the addition of ligands that targets specific components of the tumor microenvironment if such addition does not compromise the efficiency of the biotransformation process.

### 4.7. Exploiting the TME (Tumor Microenvironment)

It has been previously established that the tumor microenvironment consists of fibrous extracellular matrix (ECM) proteins, proteoglycan, growth factor receptors, and transmembrane receptors [5,21]. With the rapid growth associated with tumors, the tumor core is mostly hypoxic due to anaerobic respiration [14]. Hypoxia promotes intratumoral heterogeneity, inhibits innate and adaptive immunity, enhances metastasis, and promotes tumor resistance to ROS-generating cancer therapies (photodynamic therapy, sonodynamic therapy, chemotherapy, and radiation) thus leading to ineffective therapeutic outcomes. Besides, there is a high interstitial fluid pressure [2] within tumors that serves as a barrier to nanoparticle penetration hence drastically reducing vascular transport into the core of the tumor tissue. These obstacles make tissue penetration a daunting task for nanoparticles. To achieve adequate tumor accumulation via the EPR effect, there is a need to exploit the opportunities provided by the TME.

#### 4.7.1. Degradation of ECM to Improve EPR Effect

To improve accumulation and penetration of nanoparticles in neoplastic tissues, degradation of the fibrous ECM with hyaluronidase, an enzyme that breaks down collagen, is a strategy used in combination with other techniques to enhance the EPR effect [5]. For nanoparticles to gain access to the core of tumor tissues, it is imperative that the barriers that prevent accumulation and penetration of nanoparticles in tumors be removed. The challenge of degrading the ECM is the risk of increasing metastasis as the tumor cells become easily mobile when the ECM is compromised [5]. Phesgo^®^, a combination of two monoclonal antibodies, pertuzumab and transtuzumab for treatment of HER 2+ breast cancer, incorporates hyaluronidase as part of a tripartite strategy to target HER2 receptors in the treatment of HER2+ tumors [116]. Both transtuzumab and pertuzumab are included in this product for their capacity to actively target domains IV and II of the epidermal growth factor receptors (EGFR) respectively, that are overexpressed in several cancers like breast, cervical and intestinal cancers [117,118].

#### 4.7.2. Integrin Receptors as Target for Improving EPR

Integrin αvβ3 receptors are an established tumor-specific marker of angiogenic activity in the ECM that are overexpressed in rapidly proliferating blood vessels, playing key roles in tumor growth and metastasis [21]. Targeting integrin αvβ3 leads nanoparticles to the rapidly forming and leaky tumor vessels, thereby increasing extravasation and accumulation of nanoparticles in the TME via the EPR effect [21]. The affinity of the peptide ligand Arg-Gly-Asp (RGD) for integrin αvβ3 is well established and its use in research is richly documented [15,21]. In a published article, epirubicin loaded near-infrared (NIR) fluorescent nanoparticles assembled from multiple units of cyclic peptides, cyclo [-(D-Ala-L-Glu-D-Ala-L-Trp)2-] were decorated with RGD peptide to evaluate its targeting efficiency to integrin receptors [119]. The authors reported an increased accumulation of the nano construct in tumor tissues compared to healthy tissues. The selective binding of RGD decorated nanoparticles was attributed to the presence of integrin α_v_β_3_ receptors on the tumor cells. RGD selective binding in this study led to a substantial improvement in EPR which enhanced therapeutic efficacy.

#### 4.7.3. Exploiting Hypoxia within Tumors

Tumor tissue hypoxia can be harnessed to improve EPR in several ways. One such way is by targeting molecular markers such as phosphatidylserine (overexpressed in hypoxic regions) with Sapocin C, a lysosomal protein that binds to it specifically in hypoxic environment [120]. Selective targeting of tumor phosphatidylserine by Sapocin C spares phosphatidylserine expressed on healthy cells. By designing drug loaded nanoparticles with linkers (azobenzene or 2-nitroimidazole) that can be degraded in a hypoxic environment like tumor tissues, EPR can be greatly improved [121].The reader is referred to reviews by Milane [14,120] for details on other hypoxia based techniques.

### 4.8. The Use of EPR-Adaptive Delivery Strategies

Apart from modification of the physicochemical properties of nanocarriers, various chemical and physical approaches have also been used to modify the TME for enhanced accumulation of macromolecules via the EPR effect. Modification of the TME can be achieved using external physical or chemical delivery strategies. Chemical EPR-adaptive delivery strategies involve using EPR enhancement factors to adjust the tumor vasculature while external physical inducements typically applied are photodynamic therapy, radiation, sonoporation, and hyperthermia to enhance tumor vascular permeability [122,123]. For in-depth review of these methods, the reader is referred to: Fang [19] and Golombek [2] for PDT; Golombek et al. [2] Park et al. [124] for radiation therapy; Fang et al. [19] and Park et al. [21] for hyperthermia; and Iwanaga et al. [125], Duan et al. [126] and Theek et al. [127] for sonoporation. A recently published review dwelt extensively on physical and pharmacological strategies to improve the EPR effect and the interested reader is referred [128]

#### 4.8.1. Physical Methods

Physical techniques have been developed by various groups to enhance the EPR effect towards improving the therapeutic efficacy of nanomedicines [2,19,21,119,126,127].

##### Photodynamic Therapy (PDT)

Photodynamic therapy (PDT) as one such example refers to the treatment of tissues, typically tumors, with a photosensitizing agent, followed by activation via locally applied light of specific wavelength [129]. The principle is based on the formation of reactive oxygen species (ROS), such as singlet oxygen species which damages nucleic acids and protein resulting in apoptosis of cancer cells [2]. The decrease in mass of the tumor creates an enabling environment for accumulation of nanoparticles via EPR due to the decrease in interstitial fluid pressure and overall ECM mass. PDT is limited by penetration depth of the applied light (max. 1–2 cm), and the short migration distance of the produced oxygen radicals, and this reduces the impact on the tumor core [2]. One study utilized PDT to target tumor blood vessels by tagging photosensitizers with RGD to enable interaction with endothelial integrin. Application of PDT increased vessel permeability and consequently, enhanced EPR [130]. The authors reported markedly increased tumor accumulation of doxil^®^. Pretreatment of tumor tissues with photodynamic therapy (PDT) before administering nanomedicine has been demonstrated to improve therapeutic efficacy due to a more efficient accumulation of nanocarriers within the tumor [131]. For example, a 5-fold increase in the tumor accumulation of liposomes loaded with daunorubicin was observed in EGFR-positive A431 epidermoid carcinoma cells coexisting with a small fraction of EGFR-negative Balb-3T3 embryonic fibroblasts after pre-treatment with EGFR-targeted photodynamic therapy [31].

##### Radiation Therapy (RT)

Radiation therapy (RT) is commonly used alone or with chemotherapeutic agents in cancer treatment. Ionizing irradiation used in radiation therapy has the ability to decrease IFP (interstitial fluid pressure) by generating cytotoxic radicals leading to a decrease in cell density within tumors [132]. Ionizing radiation can also increase tumor vascular leakiness through upregulation of VEGF expression and fibroblast growth factor [124]. Taken together, the various effect of radiotherapy increases the accumulation of low-molecular-weight drugs and nanomedicine formulations in the tumor microenvironment. When radiotherapy is combined with nano-based chemotherapy, there is an increase in the anti-cancer treatment effectiveness. For instance, verteporfin-loaded nanoparticles injected into mice with rhabdomyosarcoma tumors and exposed to laser light [635 nm (0.2 mW cm^−2^) for 1 min] at 15, 30, and 60 min after injection demonstrated enhanced accumulation of nanoparticles at tumor site. The mice exposed longer to the laser light showed significantly less side effect [133]. In another instance, an analysis of the impact of nanoparticles with RGD moieties in conjunction with radiation therapy showed that treatment with radiation improved the therapeutic response to chemotherapy [119] most likely because of improved EPR.

##### Hyperthermia

The use of high temperatures (between 39 °C and 42 °C) is another EPR enhancing strategy that induces tissue ablation and promotes perfusion, vasodilation, and vascular permeability and extravasation (i.e., EPR effect) [19,21]. This high temperature strategy is referred to as hyperthermia. Hyperthermia induced by high-intensity focused ultrasound (HIFU) has been used to increase tumor perfusion and vascular permeability within neoplastic tissues [21]. It can be used to increase nanomedicine accumulation, especially in non-leaky tumors which exhibit low EPR effect [2]. Rhodamine-labelled liposomes (100 nm) demonstrated enhanced accumulation in tumor interstitium after intravenous injection into xenograft human ovarian cancer models that had been subjected to gradual temperature increase (between 39 °C and 42 °C) for one hour. The tumors were previously impervious to the rhodamine liposomes at ambient temperature [134]. The ability of HIFU to enhance the EPR effect has been evaluated in various preclinical studies and is close to clinical translation [122]. Temperature-sensitive nanoformulations e.g., ThermoDox^®^, have become the focus of new research due to the universal relevance of vascular heat shock response after exposure of vessels to increased temperature [21].

##### Ultrasonication

Ultrasonication using micro/nanobubbles as contrast agents has been utilized in imaging techniques; however, it can also be exploited to increase vessel perfusion and permeability to improve EPR effect [125]. Acoustic transducers are used to generate transverse or longitudinal waves with frequencies greater than 20 kHz, which causes nanobubble oscillation or implosion, leading to vascular cavitation that results in the extravasation of loaded nanobubbles into the tumor microenvironment [126]. Drugs may be encapsulated in the microbubble core or conjugated to the microbubble either directly or indirectly to suitable nanocarriers [135]. Other techniques involve the co-delivery of microbubbles and nanoparticles. This codelivery strategy allows the nanoparticles to take advantage of the cavitation created at the tumor site via an increase in microbubble size in response to transducers [126].

Ultrasound waves have a greater ability to penetrate tissue compared to light waves in PDT, and therefore, are more useful for deep-seated tumors [21]. Ultrasound imaging can produce sonoporation within cell membranes due to the generation of reactive oxygen species by ultrasound waves. When sonoporation is controlled with an ultrasound contrasting agent, temporary pores can be produced within the endothelial layer, causing increased vascular permeability which improves EPR [126]. Micro/nano-bubbles (MNBs), which are used as ultrasound contrast agents, have been employed to control temporary increases in tumor vascular leakiness for improved nanomedicine accumulation within tumor tissues [126,136].

**Figure 8:**
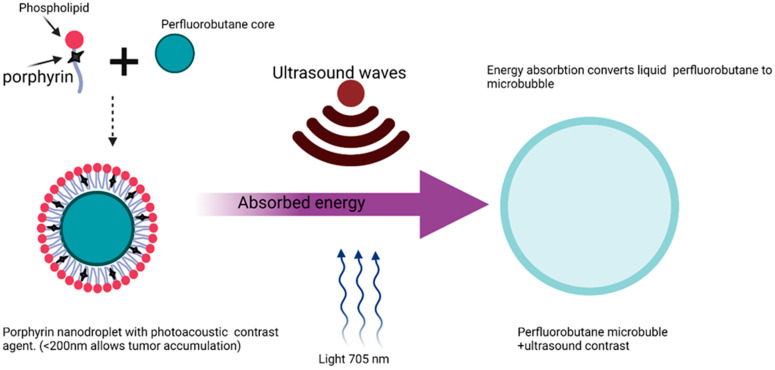
Phase conversion strategy of microbubble ultrasound-based delivery. Porphyrin nanodroplets provides photoacoustic contrast due to the strong optical absorption of porphyrin. The absorption of photons or acoustic energy induces a phase transition of perfluorobutane from liquid to gas microbubble. This system can be loaded with a payload for delivery through cavitations induced by the hyper-echogenic and nonlinear acoustic properties of microbubbles. Modified from Ref. [126] and created with BioRender.com Accessed on 15 March 2022.

When exposed to ultrasound, micro-nanobubbles undergo oscillation which generates a fluid flow that increases the permeation and accumulation of encapsulated payload in tumor tissues by the EPR effect [126]. In one report, transferrin conjugated nanobubbles were used to traffic drug loaded nanoparticles to the tumor site. Upon application of external ultrasound waves, vascular agitation by the waves led to an increase in the permeability of the nascent blood vessels, resulting in the enhanced delivery of nanoparticles to deep sites within the neoplastic tissue, thus enhancing EPR [137]. The combination of ultrasound, microbubbles, and gemcitabine was evaluated in a phase II clinical trial. The result showed that there was no added toxicity in the combined regimen, and patients tolerated an increased number of gemcitabine cycles compared with historical controls. In addition, the result demonstrated a progressive decrease in tumor size and an increase in the median survival time (17.6 months) in the experimental arm compared to the historical control arm (8.9 months) [138]. Likewise, nanoparticles loaded with doxorubicin were reported to demonstrate enhanced accumulation and distribution within tumors by low-intensity focused ultrasound [139]. Ultrasound waves can be transformed into heat energy due to the friction generated as they propagate through tissue. The heat energy produced may enhance nanocarrier extravasation in tumor tissues by altering tumor hemodynamics. Micronanobubbles have been applied for theranostic purposes by using a phase conversion strategy to deliver ultrasound aided contrast agents via the EPR effect as shown in Figure 8 [126].

#### 4.8.2. Chemical EPR-Adaptive Delivery Strategies

Chemical strategies involve modifying the blood vessels with endogenous cytokines and pharmacological agents to overcome factors associated with the poor accumulation of nanoparticles in tumor cells. These factors include poor tumor perfusion, irregular leakiness, and compressed blood vessels that together hinder the accumulation of adequate concentrations of nanocarriers at the tumor site. Several vasomodulators have been used to enhance tumor blood perfusion, with the expectation that such perfusion will translate to better drug delivery. These agents include bradykinin, tumor necrosis factor-alpha (TNFα), serotonin, and histamine [19,140]. However, toxicity from the systemic administration of cytokines has limited their application. Pharmacological agents that produce a similar vasomodulatory effect to that of cytokines are nitric oxide, angiotensin II blockers, angiotensin converting enzyme inhibitors (ACEI), and nitroglycerin [141,142,143]. The use of pharmacological agents relies on their capacity to reduce peripheral resistance, which translates to enhanced tissue perfusion. Chemical EPR-adaptive delivery strategies have been discussed in-depth by several authors and are outside the scope of this review. The interested reader is referred to reviews by Ikeda-Imafuku [128] and Ojha et al. [144] for chemical strategies to improve EPR.

## 5. Development of Rapid Quantitative EPR-Imaging Technologies

Imaging technologies which make it possible to visualize and quantify the extent of EPR-based tumor targeting can provide critical information for future nanocarrier design, predictions of nanomedicine accumulation, and in the pre-selection of patients who would respond to EPR-based therapies [5,126,145,146]. Single photon emission computed tomography (SPECT), positron emission tomography (PET), computed tomography (CT), and MNBs-enhanced (micro nanobubbles) ultrasound imaging have been used to quantify nanomedicines accumulation and distribution in human tumors [20,147]. A radiolabeled analog of a nanomedicine could be given to a cohort of patients individually, and its accumulation quantified in each patient. Patients showing evidence of accumulation (EPR positive group) could then be treated with the nanomedicine, following standard protocols, while the patients with no evidence/or suboptimal level of nanomedicine accumulation could be moved to a different type of treatment [32]. Miller and colleagues showed that ferumoxytol containing super paramagnetic iron oxide nanoparticles could be used as a substitute or companion particle to predict the extravasation, distribution, and accumulation of a PLGA-PEG-based nanomedicine in tumors [148]. More recently, a vascular multiphase tumor growth model that can pre-check the effects of EPR factors such as tumor lymphatic drainage or the size and permeability of vascular endothelial cell pores on the biodistribution of different sizes of nanoparticles in the TME was developed and tested [30].

## 6. Conclusions

The EPR effect can be considered a hallmark mechanism that differentiates a tumor from healthy tissue and exploits the anatomical and pathophysiological defects in the tumor vasculature to achieve selective anticancer nanomedicine delivery. The desire to improve therapeutic efficacy and safety of clinically relevant and newly developed antineoplastics has led to the advancement of several strategies to precisely deliver these drugs to tumors. Passive targeting strategies based on the EPR effect have shown great therapeutic potential in various preclinical animal models. However, the therapeutic outcome of passively targeted nanomedicines in clinical practice is not encouraging, mainly due to the inherent heterogeneity of the EPR effect. In addition, it has been recognized that for most actively targeted techniques, an effective EPR strategy is still a sine qua non to efficient targeting. Despite the best effort of researchers, the delivery and accumulation of nanoparticles at tumor sites remain very low. Although the notion of the poor delivery of nanoparticles with previous strategies has been disputed, and some published studies have shown tremendous improvement in terms of percent delivery, it remains to be seen if these strategies will translate to clinical success. Thus, understanding and manipulating the factors contributing to the EPR effect can further improve the selective targeting of anticancer nanomedicines to tumors.

Our review differs from a recent review of strategies to improve the EPR effect by Ikeda-Imafuku et al. [128]. In their review, they extensively explored the literature on physical and pharmacological techniques to improve EPR. While there are areas of overlap in general background information, our review focuses on molecular dynamics and challenges in the odyssey of nanomedicines and macromolecular carriers. Our goal was to explore existing and novel nanoformulation strategies that have been demonstrated to improve the EPR effect.

In this review, the critical issues that are essential to improving the EPR effect for increased nanoparticle and macromolecule accumulation at tumor sites are discussed. Since all anticancer nanomedicine delivery systems benefit from the EPR effect, passive and active targeting strategies should be combined in the design and development of nanomedicines to facilitate EPR-based tumor accumulation for better therapeutic efficacy and reduced adverse effects.

## Data Availability

Not applicable.
